# Monthly Summary

**Published:** 1879-03

**Authors:** 


### MONTHLY SUMMARY.

*Good Effects of Ligation of the Thighs in Obstinate Epistaxis.* —Gazette des Hopitaux: M. Blondeau reports the case of a gouty subject who had an attack of epistaxis in which nearly two quarts of blood was lost, and which ceased only upon the occurrence of syncope. Eight days later a second attack occurred. Injection of cold water containing perchloride of iron were first tried in vain, and a ligature was then applied tightly to the middle of the thigh. The hemorrhage ceased almost immediately, but re-appeared on the following day shortly after the ligature had been moved. The ligature was again applied with equal success, but its removal was again followed by recurrence of the epistaxis. The treatment was persevered in, and finally, after several days, the hemorrhage ceased completely. —*Louisville Medical News.*

